# Recognising the Value of Everyday Interactions in Inpatient CAMHS: Patient Thank‐You Letters as Insights Into Nursing Impact

**DOI:** 10.1111/inm.70062

**Published:** 2025-05-14

**Authors:** Sebastian Monteux, Fiona J. Stirling, Marcia Stoll, Lynne Thomas

**Affiliations:** ^1^ Abertay University Dundee UK

**Keywords:** child and adolescent mental health, everyday interactions, gratitude, mental health nursing, qualitative thematic analysis, unsolicited patient feedback

## Abstract

This study explores patient feedback through unsolicited thank‐you letters, asking: *What insights do these letters provide into impactful nursing practices in inpatient CAMHS?* Using an exploratory qualitative approach, data from two focus groups with mental health nurses (MHNs) and an online questionnaire were analysed through thematic analysis, identifying three key themes—‘*Being Present*,’ ‘*Being Skilful*,’ and ‘*Being Human*.’ Findings reveal that thank‐you letters offer unique, spontaneous insights into the aspects of nursing care young people value most, highlighting everyday interactions over structured clinical interventions. However, these relational aspects of care are often undervalued in inpatient settings. To ensure they are recognised and sustained, inpatient CAMHS should integrate thank‐you letters into staff training, reflective practice, and service development. This study underscores the need to reframe everyday nursing interactions as essential rather than incidental, recognising their critical role in therapeutic engagement and patient well‐being.

## 1 Introduction

Concern for child and adolescent mental health is an increasingly global topic of interest (Kaku et al. 2022), with recent research highlighting worsening trends worldwide (McGorry et al. 2024). In Scotland and across the UK, there is growing recognition of the need to improve the quality of Child and Adolescent Mental Health Services (CAMHS). The Scottish Government's Mental Health Strategy 2017–2027 emphasises the importance of developing a confident and skilled workforce across all four CAMHS tiers, while the NHS Long Term Plan (2019) commits to enhancing the quality of inpatient care for children and young people. Research indicates that the quality of the inpatient CAMHS experience significantly influences both short‐ and long‐term outcomes for young people (NIHR 2021). In particular, relationships within inpatient services have been identified as one of the four critical influences on young people's experiences of mental health care (NIHR 2021). Despite these efforts, persistent challenges remain in ensuring that inpatient CAMHS services effectively meet the needs of young people, highlighting the importance of research and evaluation in this setting.

Yet, as Kirk et al. (2023) highlight, efforts to improve inpatient CAMHS have had limited success. A systematic review by Staniszewska et al. (2019) found that while substantial research exists on inpatient experiences more broadly, there is little evidence on what young people themselves find most valuable. Despite the recognised importance of patient feedback, there remains a gap in how inpatient CAMHS experiences are meaningfully captured and explored. This has led to calls for broader and more nuanced methods of gathering feedback to reflect the unique relational and technical challenges inherent in inpatient CAMHS services (Clark and MacLennan 2023). Exploring alternative feedback mechanisms may therefore provide valuable insights to improve patient experiences and outcomes. Mental health nurses (MHNs), who constitute the largest proportion of the CAMHS workforce (NES 2022), play a central role in shaping inpatient experiences. However, research indicates that MHNs often feel undervalued, under‐supported, and constrained by systemic pressures, which can impact their ability to engage meaningfully with young people (Hartley et al. 2022).

MHNs working in CAMHS inpatient settings face distinct professional and personal challenges (Stirling et al. 2023), yet there are no established specialist training pathways to equip them with the necessary skills. This not only affects the experiences of service users but also impacts staff well‐being, job suitability, and retention. The Public Accounts Committee (2019, 6) has identified workforce shortages as the “biggest barrier to the government's ambitions for children and young people's mental health services.” Despite their critical role, research focused on MHNs within inpatient CAMHS remains limited. This study seeks to address this gap by examining how young people's unsolicited thank‐you letters provide insights into the aspects of nursing care they find most impactful. By highlighting young people's own words, this study prioritises patient‐led perspectives in shaping service improvements.

There is widespread agreement that patient feedback is crucial for evaluating and improving healthcare (McIntosh et al. 2024). While some feedback is collected through formal national and local surveys, the most common form received by nursing staff is unsolicited and informal (Lloyd et al. 2023). Thank‐you letters, as spontaneous expressions of gratitude, are often overlooked despite their potential to offer meaningful insights into service quality and relational care (Herbland et al. 2017; Aparicio et al. 2017; Day et al. 2020). Gillespie and Reader (2021, 484) note that this reflects a broader tendency in healthcare research to focus on “what goes wrong rather than what goes right.” Previous studies have shown that positive service user feedback can be a powerful driver for change and have called for policymakers to facilitate the collection and use of such feedback in reflective practice (Lloyd et al. 2023; Sheard et al. 2019). Notably, gratitude expressions have been linked to improved therapeutic relationships, increased staff resilience, and reduced burnout (Converso et al. 2015; Starkey et al. 2019).

The data in this study have generated two separate publications. The first paper (Stirling et al. 2023) focused on MHNs' experiences of receiving thank‐you letters and their impact on their professional role and well‐being. However, the letters also provided rich insights into young people's care experiences, which warranted a dedicated exploration. This second paper focuses on young people's perspectives, examining how their letters illuminate impactful nursing practices in inpatient CAMHS.

In summary, this study contributes to research on inpatient CAMHS and the role of MHNs in shaping patient experiences. It also expands the understanding of thank‐you letters as a valuable yet underutilised form of patient feedback. While relational care is a well‐established concept (Tolosa‐Merlos et al. 2023), these letters offer a unique perspective on the interactions that young people find most meaningful. By exploring unsolicited thank‐you letters, this study provides novel insights into the everyday nursing practices that shape young people's experiences of inpatient CAMHS.

## 2 Method

Full details of the recruitment and methods have been published previously (Stirling et al. 2023) however, a brief overview is provided below for the convenience of the reader.

### 2.1 Design

The study adopted an exploratory qualitative approach using focus groups guided by a standardised semi‐structured interview protocol. Participants were asked to bring along any thank‐you letters of thanks they had received during their employment at the YPU, and these were referenced directly during discussions. Each focus group lasted 1.5 h and had two facilitators present. This allowed one facilitator to guide the focus group discussion with the prepared interview protocol, while the other collected live process notes. A brief online follow‐up questionnaire, made up of three free‐text response questions, was used to capture any further comments and reflections from participants on the focus group experience.

### 2.2 Participants

Seven registered mental health nurses working in the same Young Persons Unit (YPU) were interviewed. Of these seven participants, six were female and one was male, four were staff nurses (Band 5), two were charge nurses (Band 6) and one had a split role in the unit (charge nurse/systemic family therapist). Years of experience as registered mental health nurses ranged from 2 to 17, and time spent working in the unit ranged from 1 to 16 years.

### 2.3 Data Collection

Research was carried out in person at the service site, a Tier 4 NHS CAMHS YPU in Scotland. Two audio‐recorded focus groups were run, one with four participants and one with three, to allow for longer discussion time, accommodate nurses' schedules, and adhere to COVID‐19 protocols in place at the time.

### 2.4 Ethical Considerations

Data were collected following both University and NHS ethical standards and confidentiality protocols.

### 2.5 Analysis

Interviews were transcribed, revealing two distinct areas of insight: nurses' experiences of receiving gratitude through thank‐you letters and patients' experiences in the YPU, as reflected in the content of these letters. This paper focuses on the latter. Thematic analysis (Braun and Clarke 2006), a method particularly well‐suited to qualitative research in health and well‐being, especially in practice‐focused studies (Braun and Clarke 2014), was employed to analyse the data. Braun and Clarke's (2006) six‐phase, data‐driven approach was used to systematically explore the data and identify key themes.

## 3 Results

From the data available three themes were identified, with a total of 13 subthemes. The categorisation and naming of these themes was informed by the researchers professional knowledge of the nursing role, linking the insights from the data to ways in which nurses can interact under an overarching concept of ‘being with’.

We acknowledge that the subthemes are overlapping and not necessarily exclusive to the themes assigned however we believe they represent distinct ways of ‘being with’ as communicated through the data. The potential for subthemes to overlap also mirrors the complexity inherent to performance of the MHN role, particularly within inpatient CAMHS settings.

An overview of the themes and subthemes is provided in Table 1.

**TABLE 1:** Overview of themes and subthemes.

Overarching theme: being with		
Theme 1: Being present	Theme 2: Being skilful	Theme 3: Being human
Being actively present Engaging in joint activities Providing a safe environment	Listening Processing feelings Unconditional Positive Regard Rapport building Learning and responding to individual preferences Holding Hope	Little things Authenticity Travelling together on a journey Humour and positivity

### 3.1 Theme 1: Being Present

The first theme captures an appreciation for nurses ability to be continuously present during the care experience. While care in the YPU is delivered by an interdisciplinary team it is the nursing staff that are there for ‘24 hours; it's being there all the time’. Being there, showing up, and choosing to stay, even in the most difficult moments:

They say Thank you for being there for me during the darkest times. From sitting through in [A&E] with me until now, I can't thank you enough. (P4)

I think that's when you start getting these kind of messages, that you think actually, what the young person's seeing is a bunch of adults who don't run away or don't react in ways that they have maybe seen being reacted to in these situations. (P5)

While the nursing role is constituted from many parts, it was this showing up consistently that mattered to young people:

That's like the key part that I drew from the card, that was what they felt was the most important stuff. Not all the extra bits and pieces that go into it, but just the being there. (P6)

They say with your help I won't ever have to go back to the place I was in. And, yeah, just thanking me for kind of being there. (P5)

Time spent together between nursing staff and young people could be orientated around an activity:

Just sitting and watching TV. (P4)

Play pool with them or just spend some time with them in the art room or something. (P1)

Or it could be a choice simply to remain physically present:

I might just feel like I'm sitting here and they're not interacting with me at all. But actually, do you know what? I'll sit here another 5–10 minutes. (P5)

Spending time with them. (P3)

Nurses' ability to be present offered young people a sense of safety and security not available to them elsewhere.

Some people find it's better here than being at home. And that's the hard part. They feel safe here. (P2)

There is also acknowledgment of the active efforts nursing staff make to facilitate that safety:

[they say] Thank you for keeping me safe when I didn't want to be. (P1)

### 3.2 Theme 2: Being Skilful

There were a number of practical skills highlighted as helpful for the young people. Listening was one of these:

They feel listened to here. (P2)

They've said, from my rants; thank you for listening to my rants. Because obviously they're distressed, and it's not judging or anything; just being there for them. (P2)

Recognition was also given to assistance in understanding and processing difficult feelings:

[they wrote] You helped me open up all the past, and now it's OK to cry. I don't think you realise how much I find it hard to cry. But with your helping hand and kindness, I got there eventually, and this might be the beginning of change for me potentially. (P7)

And she also says like she, as in me, has a voice that could calm a hurricane. (P6)

Offering unconditional positive regard and acceptance to the young people was experienced as helpful:

[they say] you've helped me be OK with myself. Sort of accept myself for being different, and that's OK. (P6)

One of mine is an ace card, and it's like thanks for believing in me. Because I think we talk a lot about, we believe in you and you deserve this. (P1)

Rapport building was recognised as the ‘quick conversations here and there’ which kept nurses and young people connected. Rapport was also facilitated by time for informal connections.

I think the nursing team gets a lot more opportunities to have those informal chats and build those informal relationships, whereas other professionals tend to be meeting a patient for a specific purpose […] you get the opportunity to build those little more natural relationships. (P4)

When rapport is successful it seems important to follow information gathered through that process with recognition and respect of young people's individual choices and preferences.

She said, Thank you for taking an interest in my religion. Her religion was obviously something really important to her, but I didn't think about it at the time. I was just trying to get to know her and just ask questions about it. But obviously that meant quite a lot to her, and I was surprised by that. (P1)

Lastly, the ability to ‘hold’ or maintain hope for the young people was considered valuable:

You always held onto the hope until I was ready to take it back. That bit about holding onto hope, I think that's definitely kind of stuck with me, and I think it's something that I'm aware that I really refer to that with other young people now about that being part of my role, or part of the team's role. Holding onto their hope for them, at times when they're maybe not able to. That's what I've started kind of taking away from it all, that's totally part of my script now in terms of working with young people. (P5)

We're going to hold onto your hope until we can give it back to you type thing. (P1)

### 3.3 Theme 3: Being Human

The third theme captures elements of everyday or simple actions, the ‘little things’ which nursing staff seemed surprised made a difference or were recognised by the young people.

[It said] Thanks for always tasting the cakes I made. (P5)

So, one of the things he really enjoyed was accents. We used to sit and put on accents to each other. But in the conversations, we would open up more because we were doing a silly voice. So, he has made reference to say like, I think we could safely pass as Australian. (P6)

I've kind of reduced that feeling of needing to feel like I'm doing something really massive or really amazing or the best bit of nursing you've ever seen. It's all the little bits that seem to actually build up to that, and just being OK with that. (P5)

Little things being recognised was connected to the authenticity or realness of the nurse—how willing they were to bring their individual personality to their role, or to behave as ‘human’.

Meeting somebody on a human level, just being with them. (P5)

So, for me it's more like, don't feel like you have to be someone else. Like, use your own personality traits, because there is always someone that will attach to them, if that makes sense. (P4)

Just be natural to yourself. Like, you have to use your own character; you can't be something that you're not. (P4)

You know, the little kind of personal bits that would show the human bit of us. I think that is definitely what the young people connect with. (P5)

Being genuine, isn't it? It's not being fake at all. There's no point; they'll see right through it. (P2)

All the stuff that's fed back here is about me having these weird little—I don't know if idiosyncrasies is the right word, but—little weird things that we do together that obviously they have found meaningful. (P4)

Being human facilitated a sense of being alongside the young people, travelling together on their journey through the unit and building a sense of companionship or friendship.

[the card is] highlighting that time we went through together. (P4)

They've reflected back on the journey that we had and about their experiences and times that we've shared together. (P6)

It was not necessarily deeply therapeutic; it was more, she describes it as a friendship. (P6)

Lastly, humour and positivity were highlighted as meaningful, and ‘banter’ allowed this humour to be traded back and forth between the nurses and young people.

[they wrote] thank you for helping me in my recovery and for making me laugh and making me smile. (P5)

[she wrote] I hope this gives you an insight into how much of an amazing nurse you are. Really do appreciate your input and think you're quite the star. I'll remember our chats and laughter forever, and your positivity too. So, for all of the above and so much more, I say a huge thank you. (P7)

Sometimes they write really stupid things. There's one I'm going to read; it's just silly. Because I'm vegetarian, we used to speak about chicken. She says, ‘I really hope you have a chicken burger one day’. So, she's using banter. (P2)

I've got some [thank you cards where] it's like, when I have been like silly and talk about banter, I suppose. Rather than just being more serious all the time. (P1)

## 4 Discussion

**FIGURE 1:**
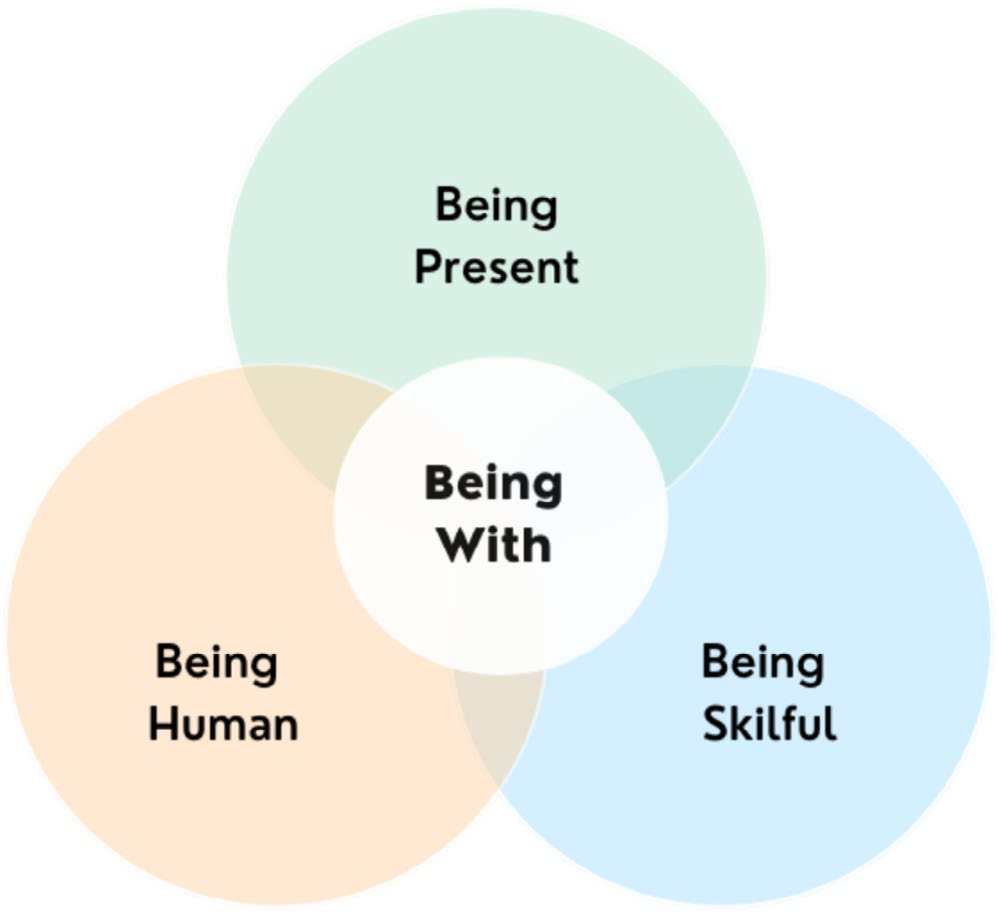
Overarching theme of ‘being with’.

This study explored unsolicited patient feedback in the form of thank‐you letters received by nursing staff in a CAMHS inpatient unit. These letters offer valuable insights into the aspects of nursing care that young people value, revealing a central, overarching theme of “*being with”* as fundamental to MHN practice and most valued in inpatient CAMHS settings (see Figure 1).

The first theme, “*Being Present*”, underscores the importance of nurses' continuous and consistent presence in everyday interactions, which provides a sense of safety and security through common acts like sitting with patients or engaging in shared activities. This presence reassures young people that they are supported and not alone, especially during particularly difficult times. The second theme, “*Being Skilful*”, focuses on the practical MHN skills of listening, understanding, and maintaining hope for patients. These skills appear to help young people process their emotions, feel accepted, and develop trust in their caregivers. The final theme, “*Being Human*”, emphasises the impact of authentic, everyday interactions such as small gestures, humour, and displays of individual personality that foster a sense of mutual human connection. These seemingly minor actions help to create a shared journey, making the care experience more personal and meaningful for young people admitted to inpatient CAMHS.

Notably, the nurses interviewed used the word “just” to preface such “simple” everyday actions 14 times (e.g., “just sitting,” “just being there”), which demonstrates how small or routine actions can be undervalued—even by those performing them. By examining the content of thank‐you letters, this study highlights that young people themselves recognise and appreciate these acts, reinforcing the importance of understanding patient perspectives in shaping service improvement.

This study contributes to existing research by highlighting that these everyday interactions are not merely incidental, but rather central to what patients value in relational practice, directly shaping their overall experience of care. To explore how inpatient settings might apply these insights to improve practice, we connected them to the established concepts of “hanging in and hanging out” and “mattering” as essential aspects of impactful nursing practices in inpatient CAMHS settings. Thank‐you letters provide an underutilised yet important resource for recognising and reinforcing these relational practices, offering firsthand insight into young people's experiences of inpatient care.

### 4.1 ‘Hanging in and Hanging out’: Everyday Care as a Cornerstone of CAMHS Nursing

In MHN, particularly within inpatient CAMHS, care is embedded in the daily relational interactions between nurses and patients. This study aligns with previous research findings that relationships within inpatient CAMHS are “essential, complicated and unique” (NIHR 2021, 19). In this setting, where the “little things” take on heightened significance, these everyday elements of care become more than mere tasks; they appear to be integral to the patient experience. To ensure this practice is sustained, institutional structures should explicitly acknowledge and support time for these interactions within daily clinical routines. Policies that measure and prioritise relational work as a key outcome could help shift the focus away from purely task‐based care.

This dynamic process, reported by young people and nurses as a mutual experience, underscores the importance of “simply relating” and signals to the young person that they are valued and “noticed” beyond their diagnosis or “challenging” moments. For nurses, this often means persisting—hanging in—even when challenges arise.

“Hanging in” has been described as a defining characteristic of effective care when working with children and young people. It involves patience, overcoming frustration, and remaining engaged when times are difficult, particularly when working with individuals who have complex care needs and/or a history of negative care experiences (Garfat et al. 2018).

However, this approach also relies on “hanging out”—spending time with patients beyond the more structured, task‐oriented clinical work typically associated with inpatient settings. In this study, patient thank‐you letters clearly illustrated how these everyday moments shaped their experience, reinforcing that simply “being present” during shared activities had a profound effect. While these moments may appear insignificant, young people report that engaging in everyday activities—such as watching TV or playing pool—creates a sense of connection and improves their overall experience.

During such moments, personal connection, shared experiences, and humour are exchanged, fostering a sense of relational closeness (Hartley et al. 2022). Previous research has likened these everyday interactions to “rituals” of care—small but powerful gestures that create a sense of normalcy and mutual experience between staff and patients (Fulcher 2003). These moments allow relationships to shift from structured nurse–patient interactions to a shared human connection within a “lifespace” (Monteux and Monteux 2020). This study reinforces the idea that impactful nursing is built on these everyday relational moments rather than solely on clinical interventions.

A lifespace approach is one where “close and effective personal/professional relationships emerge in the course of everyday encounters, through being with another person in naturalistic situations” (Smith 2012, 51). This approach emphasises what takes place in the “other 23 hours” of inpatient care (Treischman et al. 1969)—when staff and the young people are not engaged in formal treatment or therapy—as just as important as the technical interventions themselves. In this context, “being human” is experienced through the development of meaningful relationships in everyday care moments.

Previous research examining relationships within CAMHS inpatient services suggests that all parties (staff, young people, and carers) “bring their human selves to the relationship” as a key factor in effective MHN (Hartley et al. 2022, 12). This study builds on these findings by highlighting that it is the everyday moments—rather than just structured therapeutic interventions—that young people explicitly value and remember.

It appears, then, that the young people who sent thank you letters to nurses experienced the space through everyday interactions, and that these interactions provided a recognition of shared humanity and common interests. Spending time within the context of the young person's lifespace created opportunities for them to feel accompanied in a ‘human’ rather than in a strictly therapeutic sense‐ a mutual rather than a purely clinical relationality. Engaging at this level of relational practice however, requires great skill, motivation, and courage, and calls for environments that foster genuine connections and shared understanding (Monteux and Monteux 2020).

This was highlighted in the present study as ‘being skilful’ in relational care, an element of MHN highly valued by young people. By prioritising these everyday interactions, inpatient CAMHS services can foster a therapeutic environment where young people feel genuinely cared for, which has been associated with increased trust and improved overall well‐being (Tolosa‐Merlos et al. 2023). These relational moments not only enhance the immediate care experience but also contribute to long‐term engagement with mental health support, ultimately improving patient outcomes.

### 4.2 Mattering: Recognising the Significance of Everyday Nursing Practices

The concept of “Mattering” originates from efforts to understand the significance of social connections and focuses on how people perceive their sense of worth and impact within their relationships and communities (Rosenberg and McCullough 1981). Mattering is deeply connected to mutual relationships as it emphasises the idea of both giving and receiving in feeling valued. In mutual relationships, people experience a sense of mattering when they feel valued and recognised by others whilst also having the opportunity to contribute something meaningful in return (Flett 2022).

This study reinforces the idea that mattering is at the heart of impactful inpatient mental health nursing and that everyday interactions are fundamental to fostering this sense of significance for young people. In ‘being present,’ nurses demonstrated consistency in being there for young people, even during tough moments, sending a strong message that these young people matter. The reliable presence of nurses creates a sense of safety and dependability, reinforcing that the young people's well‐being is a priority. Their presence signals that the young people's lives are valued, particularly when young people express how much it meant to them that someone stayed with them through their darkest times.

By ‘being skilful’—through listening, understanding emotions, and offering acceptance—nurses show young people that their thoughts, feelings, and experiences are valued and meaningful. The ability to hold hope for young people when they are unable to do so themselves reinforces the idea that their future and potential still matter, even when they might feel hopeless.

In essence, the nurses' actions across all themes in this paper validate young people's sense of mattering by making them feel seen, heard, and valued. These everyday interactions serve as the foundation for relational nursing practice, shaping the overall experience of care and reinforcing the importance of human connection in mental health nursing (MHN). When young people feel they matter, they are more likely to engage with their care, build positive relationships with staff, and develop a stronger sense of self‐worth (Marshall et al. 2010).

This study highlights that relational nursing practices—particularly through everyday interactions—play an important role in fostering mattering, which is associated with improved emotional resilience, reduced self‐harm, and better long‐term mental health outcomes (Flett 2022; Watson et al. 2022).

### 4.3 Challenges

Despite the potential benefits outlined in ‘being present,’ ‘hanging in/out,’ and ‘mattering,’ these practices are not without challenges for MHNs in inpatient CAMHS settings. As Hartley et al. (2022) point out, such work requires staff with specific personal qualities who are given the time to engage with patients. Striking a balance between personal and professional boundaries can elicit additional emotional challenges and require ‘authentic vulnerability’ (Hartley et al. 2022; Stirling et al. 2023).

Since this study highlights that everyday interactions are central to what young people value in relational nursing care, being fully present in these moments of connection is essential but also highly demanding (Hartley et al. 2022). Ensuring success requires adequate staffing, time, and training (NIHR 2021), with staff receiving the provisions and resources necessary to spend time with patients in non‐clinical contexts. This highlights an institutional responsibility to prioritise everyday care interactions by embedding protected time for engagement into job structures, rather than relying on individual nurses to find “extra” time within already demanding workloads.

## 5 Conclusion

This study highlights how unsolicited thank‐you letters from young people in inpatient CAMHS provide unique insights into the nursing care they found most meaningful. The themes of ‘Being Present,’ ‘Being Skilful,’ and ‘Being Human’ illustrate how small yet significant everyday acts foster safety, trust, and connection, reinforcing the importance of both clinical skills and personal engagement in therapeutic relationships.

Thank‐you letters not only highlight valued aspects of care but also serve as tangible reflections of nursing impact. Systematically incorporating these letters into professional reflection, training, and supervision could help sustain relational care practices. Regularly reviewing them in team discussions would reinforce their importance, ensuring that these moments are acknowledged and integrated into service structures.

Further research could explore the role of thank‐you letters in service evaluation, identifying systemic strengths and areas for improvement beyond individual feedback. Embedding relational care into professional development and institutional priorities would help shift perceptions so that these interactions are recognised as essential rather than informal. Creating environments that support meaningful engagement between nurses and young people can enhance therapeutic relationships and improve patient experiences in inpatient CAMHS.

## 6 Relevance for Clinical Practice

These findings provide valuable insight into relational practice from young people's perspectives, emphasising the importance of everyday interactions in shaping positive inpatient CAMHS experiences. Thank‐you letters offer a critical but often overlooked source of feedback that can enhance understanding of young people's experiences beyond structured patient surveys or complaint mechanisms. Unlike formal evaluations, these letters capture spontaneous expressions of gratitude, offering a richer view of the relational aspects of care that young people find most impactful.

This study underscores that what young people appreciate most in relational nursing is not grand gestures or structured clinical interventions, but seemingly small, everyday moments of connection. Exploring how thank‐you letters are kept, displayed, or shared within institutional settings could provide further insight into their role in reinforcing relational care.

Prioritising presence through simple acts such as sitting together or sharing activities should be integrated into training, supervision, and service expectations as a core component of therapeutic care, rather than being treated as an informal practice. These everyday interactions offer emotional support and reassurance, while humour, shared activities, and casual conversations help foster deeper connections and a greater sense of mattering.

Given that patient thank‐you letters frequently highlight the significance of these small yet powerful interactions, CAMHS services should establish structured opportunities for staff to reflect on and learn from such feedback. For example, regular team discussions on patient gratitude could reinforce the value of relational work, support staff morale, and inform service improvements.

Embedding these practices requires institutional change. Staff need protected time for patient interaction beyond clinical tasks, ensuring that relational care becomes an integral part of daily practice rather than something that happens informally or in “spare time.” This calls for workforce strategies that incorporate relational care into staffing models, along with leadership initiatives that prioritise these interactions in performance reviews and team development.

Inpatient CAMHS should also encourage structured reflection on patient feedback, such as thank‐you letters, to drive continuous improvement. These letters provide direct insight into what young people value, and incorporating unsolicited patient gratitude into service evaluations, staff appraisals, and policy reviews can lead to meaningful enhancements in MHN practice.

## Ethics Statement

Full ethical approval was granted by Abertay University. Reference: EMS2511.

## Conflicts of Interest

The authors declare no conflicts of interest.

## Data Availability

The data that support the findings of this study are available on request from the corresponding author. The data are not publicly available due to privacy or ethical restrictions.
